# A Dose- rather than Delivery Profile–Dependent Mechanism Regulates the “Muscle-Full” Effect in Response to Oral Essential Amino Acid Intake in Young Men^1^,^2^

**DOI:** 10.3945/jn.114.199604

**Published:** 2014-12-10

**Authors:** William Kyle Mitchell, Beth E Phillips, John P Williams, Debbie Rankin, Jonathan N Lund, Kenneth Smith, Philip J Atherton

**Affiliations:** 3 Clinical, Metabolic, and Molecular Physiology, MRC–Arthritis Research UK Centre of Excellence for Musculoskeletal Ageing Research, School of Medicine, University of Nottingham, Derby, United Kingdom; and; 4 Departments of Surgery and; 5 Anaesthesia, Royal Derby Hospital, Derby, United Kingdom

**Keywords:** muscle protein synthesis, nutrition, essential amino acids, skeletal muscle, blood flow, anabolic signaling, muscle-full

## Abstract

**Background:** The anabolic response of skeletal muscle to essential amino acids (EAAs) is dose dependent, maximal at modest doses, and short lived, even with continued EAA availability, a phenomenon termed “muscle-full.” However, the effect of EAA ingestion profile on muscle metabolism remains undefined.

**Objective:** We determined the effect of Bolus vs. Spread EAA feeding in young men and hypothesized that muscle-full is regulated by a dose-, not delivery profile–, dependent mechanism.

**Methods:** We provided 16 young healthy men with 15 g mixed-EAA, either as a single dose (“Bolus”; *n* = 8) or in 4 fractions at 45-min intervals (“Spread”; *n* = 8). Plasma insulin and EAA concentrations were assayed by ELISA and ion-exchange chromatography, respectively. Limb blood flow by was determined by Doppler ultrasound, muscle microvascular flow by Sonovue (Bracco) contrast-enhanced ultrasound, and phosphorylation of mammalian target of rapamycin complex 1 substrates by immunoblotting. Intermittent muscle biopsies were taken to quantify myofibrillar-bound ^13^C_6_-phenylalanine to determine muscle protein synthesis (MPS).

**Results:** Bolus feeding achieved rapid insulinemia (13.6 μIU · mL^−1^, 25 min after commencement of feeding), aminoacidemia (∼2500 μM at 45 min), and capillary recruitment (+45% at 45 min), whereas Spread feeding achieved attenuated insulin responses, gradual low-amplitude aminoacidemia (peak: ∼1500 μM at 135 min), and no detectable capillary recruitment (all *P* < 0.01 vs. Bolus). Despite these differences, identical anabolic responses were observed; fasting fractional synthetic rates of 0.054% · h^−1^ (Bolus) and 0.066% · h^−1^ (Spread) increased to 0.095% and 0.104% · h^−1^ (no difference in increment or final values between regimens). With both Spread and Bolus feeding strategies, a latency of at least 90 min was observed before an upswing in MPS was evident. Similarly with both feeding strategies, MPS returned to fasting rates by 180 min despite elevated circulating EAAs.

**Conclusion:** These data do not support EAA delivery profile as an important determinant of anabolism in young men at rest, nor rapid aminoacidemia/leucinemia as being a key factor in maximizing MPS. This trial was registered at clinicaltrials.gov as NCT01735539.

## Introduction

Postprandial plasma aminoacidemia is a fundamentally anabolic stimulus that switches whole-body protein balance from negative to positive. A major component of this is driven by increases in skeletal muscle protein synthesis (MPS)^6^ (1–3). Increases in MPS in response to essential amino acids (EAAs) are dose dependent up to EAA and protein intakes of ∼10 and ∼20 g, respectively, approximately doubling postabsorptive MPS rates in young adults (4–6). There is also a restricted temporal profile by which EAAs achieve anabolism; after a latency of ∼1 h after intake of EAA/protein, MPS rates are elevated for ∼1.5 h before returning to fasting levels. Because this occurs even in the face of continued availability of circulating EAAs (7, 8), this has been termed the “muscle-full” phenomenon (9).

Reaching a plateau in the EAA dose and MPS rate response curve and a limited time-window for stimulated MPS rates dictate that provision of a single bolus of EAAs/high-quality digestible protein must only possess finite anabolic efficacy. This is in keeping with the observation that a meal containing a generous serving of protein achieves no additional increase in MPS over and above that derived from a moderate serving (i.e., a 113- vs. a 340-g steak) (10). Rather, it could be speculated that the anabolic properties of a specified amount of EAAs/protein may instead be dependent on the profile of their delivery/appearance. For example, some authors suggested that rapid aminoacidemia associated with the digestion properties of certain protein sources (e.g., whey) support greater anabolism even when approximately matched in terms of amino acid composition (11–15). If such relations between EAA delivery/appearance and ensuing anabolism could be forged, this would be important for designing optimal nutritional strategies for muscle maintenance (e.g., clinically) or to maximize exercise-induced anabolism (12, 16–18).

Determination of such “optimal” feeding regimes also demands knowledge of the significance, or otherwise, of key elements of the postprandial nutritional/endocrine axis. This includes establishing links between the profiles of plasma aminoacidemia (12), plasma insulinemia, muscle microvascular perfusion (19–21), and activity of intramuscular “anabolic signaling,” all in the context of muscle anabolism. Therefore, the aim of the present study was to determine each of these responses in healthy young individuals provided with the same volume of EAAs in 2 distinct fashions, either as *1*) a single, maximally effective (1 × 15 g) EAA bolus (“Bolus”) or *2*) as small (4 × 3.75 g), evenly spread amounts (“Spread”). The basis of this design was to address our hypothesis that the onset of a dose-dependent muscle-full state would dictate there being no anabolic advantage to providing EAAs as Bolus vs. Spread, despite marked differences in plasma aminoacidemia, insulinemia, and anabolic signaling profiles.

## Methods

### 

#### Study design.

**FIGURE 1:**
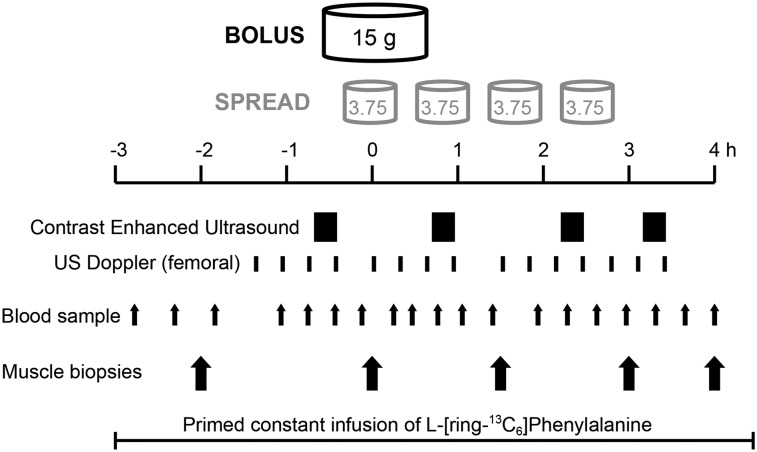
Acute study protocol. During a constant primed infusion of ^13^C_6_-phenylalanine, 15 g EAAs were ingested as a single Bolus or in 4 Spread fractions at 45-min intervals. Contrast-enhanced ultrasound, Doppler measurement of femoral flow, blood samples, and muscle biopsies were taken as indicated. EAA, essential amino acid; US, ultrasound.

Ethical approval was obtained from the University of Nottingham Medical School Ethics Committee (United Kingdom), with all studies conducted in accordance with the Declaration of Helsinki and preregistered (www.clinicaltrials.gov; registration NCT01735539). Healthy young men were recruited by response to poster advertisement/demographically targeted letters. All recruits (who were recreationally active) were studied after an overnight fast and were asked to refrain from heavy exercise for 48 h before the study. On the morning of the study (0800 h), subjects had a 18-g cannula (Vygon) inserted into the dorsum of the left hand for a primed (0.4 mg · kg^−1^) constant infusion (0.6 mg · kg^−1^ · h^−1^) of l-[ring-^13^C_6_]-phenylalanine (Cambridge Isotopes) tracer. Blood samples and muscle biopsies were taken according to the protocol (**Figure 1**). Arterialized venous blood was sampled via a retrograde 16-g intravenous cannula placed in the dorsum of the right hand, with the hand warmed to 55°C (22). Paired femoral venous samples were taken from a single lumen 14-g catheter (Vygon) sited in the right common femoral vein. Blood samples were drawn before feeding and at 15, 25, 45, 65, 80, 115, 135, 155, 175, 195, 215, and 235 min after commencement of feeding. Muscle biopsies were taken intermittently from *Musculus Vastus lateralis* using the conchotome technique (23) after infiltration of 5 mL 1% lignocaine. Muscle was washed in ice-cold saline, and visible fat and connective tissue removed before being frozen in liquid nitrogen and stored at −80°C until analysis. Biopsies were taken 1 and 3 h after commencement of the tracer to permit assessment of basal (postabsorptive) MPS. Subjects were then provided with 15 g mixed EAAs [l-histidine, 1.21 g; l-isoleucine, 1.73 g; l-leucine, 3.59 g; l-lysine, 3.07 g; l-methionine, 0.95 g; l-phenylalanine, 0.91 g; l-tryptophan, 1.13 g; l-threonine, 0.48 g; and l-valine 1.86 g; proportions were representative of muscle protein (24)] in aqueous solution (250 mL), provided in either a single dose (Bolus; *n* = 8) or in 4 equal fractions ingested at 45-min intervals (Spread; *n* = 8). Subsequent biopsies at 90, 180, and 240 min after the first feeding allowed assessment of MPS across and within the intervening periods. After the study, cannulas were removed and the subjects fed and monitored for 30 min before departure. A schematic of the study protocol is shown in Figure 1. Subject demographic characteristics are shown in **Table 1**. This was a parallel-arm, 2-group superiority trial with a 1:1 allocation ratio achieved by alternate allocation. All laboratory analyses were performed blinded to allocation. Studies were undertaken at the Royal Derby Hospital, Derby, United Kingdom, December 2011 to December 2012. The primary outcome measure was fractional synthetic rate (FSR) of myofibrillar protein synthesis (MPS) during the 6-h acute study assessed by stable isotope incorporation. Secondary outcomes were femoral blood flow and leg muscle microvascular changes as assessed by Dopper and contrast-enhanced ultrasound (CEUS), respectively. Power calculation dictated that the smallest number of subjects needed to detect (with 80% confidence, 5% significance level) a difference of 10% between groups was 8.

**TABLE 1:** Characteristics of the young men enrolled in the study^1^

	Bolus (*n* = 8)	Spread (*n* = 8)	*P*
Age, y	19.7 ± 0.5	21.5 ± 1.1	0.14
Height, cm	180 ± 1.5	180 ± 2.4	0.84
Weight, kg	74.0 ± 2.6	74.5 ± 2.4	0.91
BMI, kg/m^2^	22.9 ± 0.7	22.9 ± 0.5	0.98
ASMI,^2^ kg/m^2^	8.13 ± 0.10	8.44 ± 0.27	0.30

1 Values are means ± SEMs.

2 ASMI, appendicular skeletal muscle mass index (limb lean mass · height^−2^).

#### Measurement of plasma amino acid and insulin concentrations.

Venous plasma insulin concentrations were measured by using a high-sensitivity human insulin ELISA assay (DRG Instruments GmbH). Incremental AUC analysis estimated the total insulin response to feeding and was calculated for each individual with a baseline equal to the mean of fasting, +155 min, and +195 min insulin concentrations. Arterialized plasma amino acid concentrations were measured (Biochrom 30). Incremental AUC analysis estimating postprandial EAA and leucine exposure was calculated above a baseline of the mean of 2 postabsorptive measures.

#### Measurement of leg blood flow and muscle microvascular blood flow.

CEUS was used to measure microvascular blood volume (MBV) as previously described in detail (21). Repeated high mechanical-index flash/replenishment cycle recordings were made during intravenous infusion of Sonovue microbubble contrast agent (Bracco) and used to estimate changes in thigh MBV between recording periods indicated in Figure 1. Leg blood flow (LBF) was measured by using Doppler ultrasound, with a linear 9- to 3-MHz probe positioned over the origin of the left common femoral artery. LBF was estimated as the product of vessel cross-sectional area and mean velocity over 6 cardiac cycles. Mean LBF was calculated at a time corresponding to each CEUS recording episode on the basis of 3 such recordings made on each of 4 occasions distributed across the study day, as indicated in Figure 1 (i.e., flow during 72 cardiac cycles contributed to a mean leg flow measure). Each individual’s LBF was standardized to his own fasting LBF and normalized to leg lean tissue mass as assessed by DXA (Lunar Prodigy II; GE Medical Systems).

#### Immunoblotting.

Immunoblotting was performed on ∼30 mg of muscle as previously described (25) by using primary antibodies against ribosomal protein S6 Kinase 1 (p70S6K1)^Thr389^, protein kinase B (AKT)^Ser473^, eukaryotic elongation factor 2 (eEF2)^Thr56^ (New England Biolabs), and eukaryotic translation initiation factor 4E-binding protein 1 (4EBP1)^Thr 65/70^ (Santa Cruz Biotechnology), incubated in HRP-conjugated secondary antibody (New England Biolabs). Membranes were exposed to chemiluminescent HRP substrate (Millipore) and bands quantified by Chemidoc XRS (BioRad). All signals were within the linear range of detection (i.e., not saturated), and protein loading anomalies were corrected to Coomassie-stained membranes—a proven robust method for normalization in comparison to traditional loading controls (25). Phospho-data were not normalized to pan-antibodies because this method introduces more variation with poor stripping efficacy and the need to run independent gels. Moreover, EAA feeding is not known to acutely regulate the abundance of AKT–mammalian target of rapamycin complex 1 (mTORc1) signals. Blot data were analyzed by using peak density.

#### Muscle protein–bound and intramuscular free phenylalanine enrichment.

Myofibrillar proteins were separated, hydrolyzed, and derivatized by using our standard techniques (9, 26). The labeling of l-[ring-^13^C_6_] phenylalanine in myofibrillar protein was determined by GC-combustion-isotope ratio MS (Delta plus XP; Thermo Fisher Scientific), as previously described (27). Muscle intracellular phenylalanine enrichment was measured by GC-MS (MD 800; Thermo Fisher Scientific) after precipitation of the sarcoplasmic fraction and purification of the aqueous supernatant using Dowex H^+^ resin, as its *tert*-butyldimethylsilyl derivative. ^2^H_2_ phenylalanine was added to the intact muscle as an internal standard.

#### Rates of muscle protein synthesis (MPS).

The FSR of myofibrillar protein was calculated from the increase in incorporation of l-[ring-^13^C_6_]-phenylalanine between subsequent muscle biopsies. Muscle intracellular phenylalanine, the average of 2 biopsies, was used as a surrogate of phenylalanyl-t-RNA labeling (i.e., the immediate precursor for protein synthesis) (28). The FSR was calculated by using the standard precursor-product method; fractional protein synthesis (% · h^−1^):
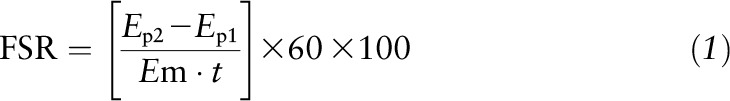


where *E*_p1_ and *E*_p2_ are the enrichments of bound l-[ring-^13^C_6_]-phenylalanine in 2 sequential biopsies, *t* is the time interval between 2 biopsies, and *E*m is the mean l-[ring-^13^C_6_]-free phenylalanine enrichment in the intramuscular pool. To offset a potential decrease in enrichment in plasma and thence intramuscular labeling with feeding, 6% of phenylalanine in the mixture was provided as l-[ring-^13^C_6_]-phenylalanine to maintain steady state labeling.

#### Statistical analyses.

Data are presented as means ± SEMs after D’Agostino and Pearson omnibus normality testing. Demographic and anthropometric comparisons between groups were determined by unpaired *t* tests. Differences for all analyses were detected by repeated-measures ANOVA and located with Bonferroni post tests by using GraphPad Prism version 5 and exact *P* values calculated by using GraphPad Quickcalcs (both GraphPad Software). A *P* value of <0.05 was considered significant. Normalization of EAA, insulin, and FSR data to 100% data span was performed by subtracting the group mean at the nadir from every individual recording before dividing each by the group mean at the maximal time point, permitting presentation on the same axis.

## Results

### 

#### Plasma concentrations of EAAs.

**FIGURE 2:**
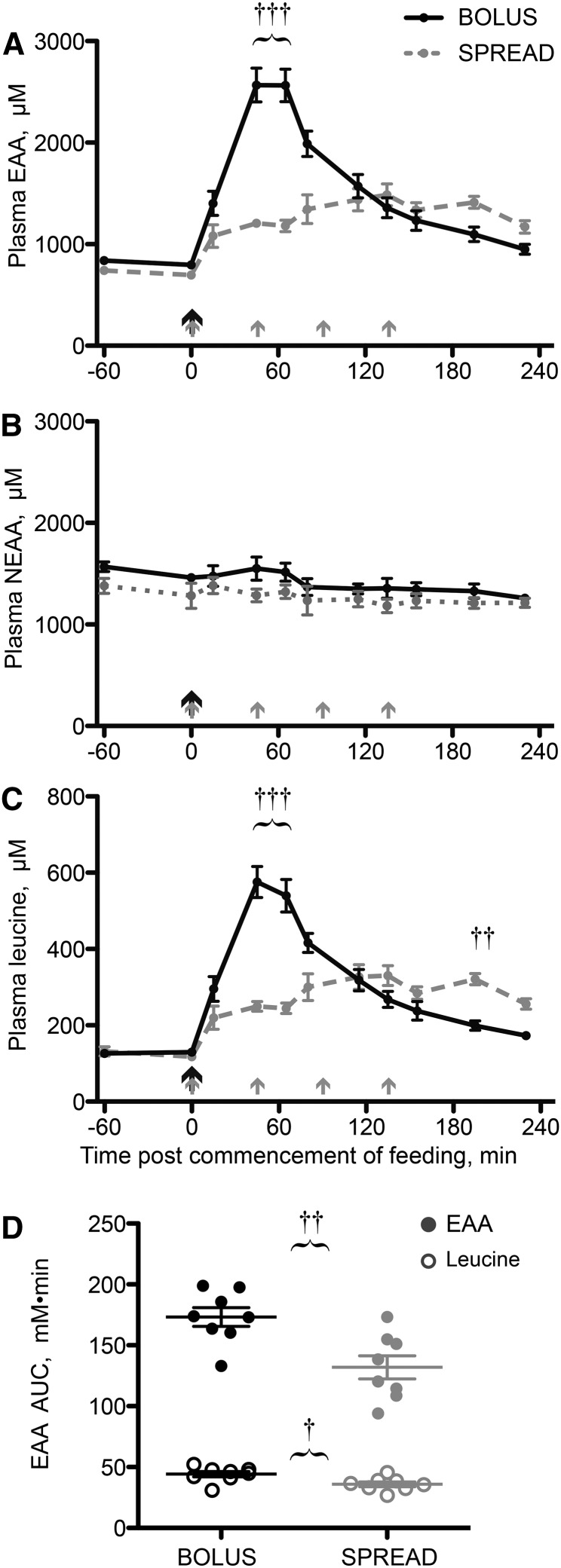
Plasma EAA (A), NEAA (B), and leucine (C) concentrations and total EAA and leucine AUCs (D) in young men after consumption of 15 g of mixed-EAA doses by Bolus or Spread treatment. The black arrows represent ingestion of 15 g EAAs once and the gray arrows represent ingestion of 3.75 g EAAs 4 times. Values are means ± SEMs, *n* = 8, or individual data points (D). ^†,††,†††^Different from Spread at that time: ^†^*P* < 0.05, ^††^*P* < 0.01, ^†††^*P* < 0.001. EAA, essential amino acid; NEAA nonessential amino acid.

After oral EAA Bolus feeding, plasma EAA concentrations increased sharply, peaking between 45 and 65 min postfeeding (+240%; *P* < 0.001 vs. fasting). This was in contrast to Spread feeding, which provided a gradual and low-amplitude essential aminoacidemia (peak: +110% at 135 min; *P* < 0.001 vs. fasting) (**Figure 2**A). Plasma non-EAA concentrations changed little across the study period and at no time differed between treatments (Figure 2B). Plasma leucine concentration changes reflected the general EAA pool (Figure 2C). AUC analyses showed greater total EAA (Bolus: 173 ± 8 mmol · L^−1^ · min; Spread: 132 ± 10 mmol · L^−1^ · min; *P* = 0.004) and leucine (Bolus: 44.2 ± 2.3 mmol · L^−1^ · min; Spread: 35.9 ± 2.0 mmol · L^−1^ · min; *P* = 0.0152) exposure, above baseline, during the feeding period (Figure 2D).

#### Plasma concentrations of insulin.

**FIGURE 3:**
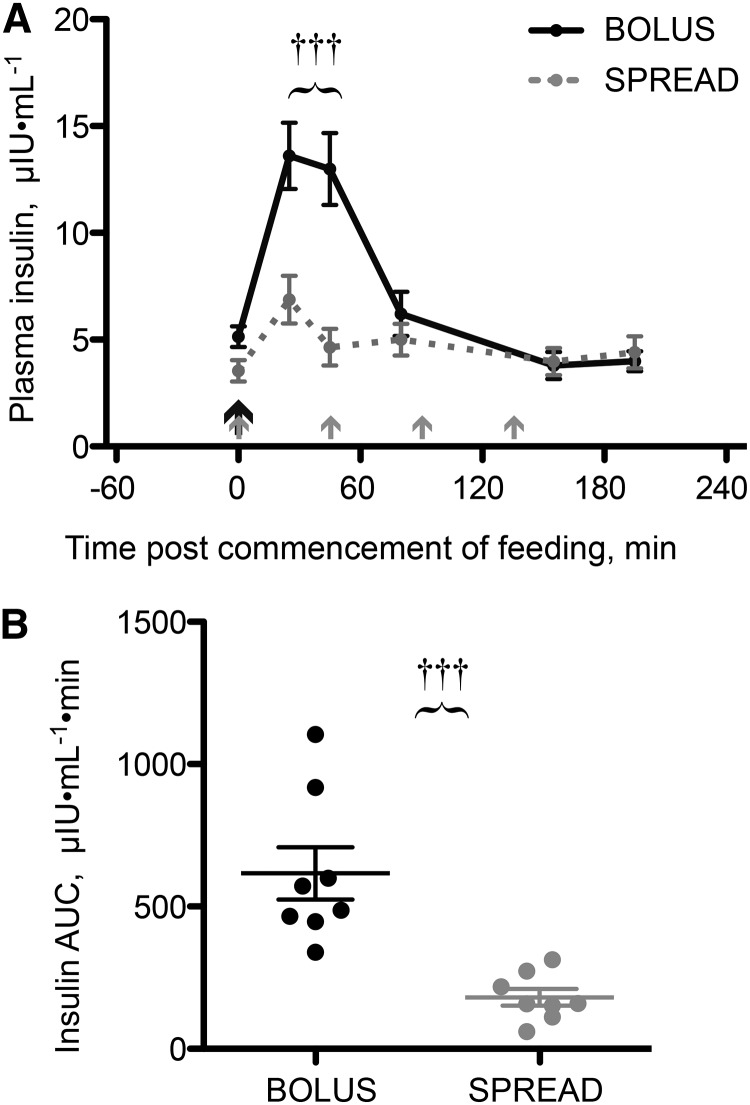
Plasma insulin concentrations (A) and total insulin expose above basal by AUC (B) in young men after consuming 15 g of mixed-EAA doses by Bolus or Spread treatment. The black arrow represents ingestion of 15 g EAAs once, and the gray arrows represent ingestion of 3.75 g EAAs 4 times. Values are means ± SEMs, *n* = 8, and individual data points (B). ^†††^Different from Spread, *P* < 0.001. EAA, essential amino acid.

Fasting plasma insulin concentrations were similar between groups (Bolus: 5.14 ± 0.5 μIU · mL^−1^; Spread: 3.54 ± 0.6 μIU · mL^−1^; *P* = 0.26). With Bolus feeding, plasma insulin was elevated (+165% at 25 min after feeding and +153% at 45 min; both *P* < 0.0001) but returned to basal concentrations 80 min postfeeding and remained at these concentrations for the rest of the study. Spread feeding achieved an attenuated insulin response (+97% at 25 min; *P* = 0.002), returning to basal concentrations thereafter (**Figure 3**A). Plasma insulin was thus different between groups at 25 and 45 min after the commencement of feeding (*P* < 0.001). The area under the plasma insulin time curve, above baseline for each individual, was 3.4 times greater with Bolus than with Spread feeding (*P* = 0.0003; Figure 3B).

#### Oral EAAs induced changes in LBF and muscle MBV.

**FIGURE 4:**
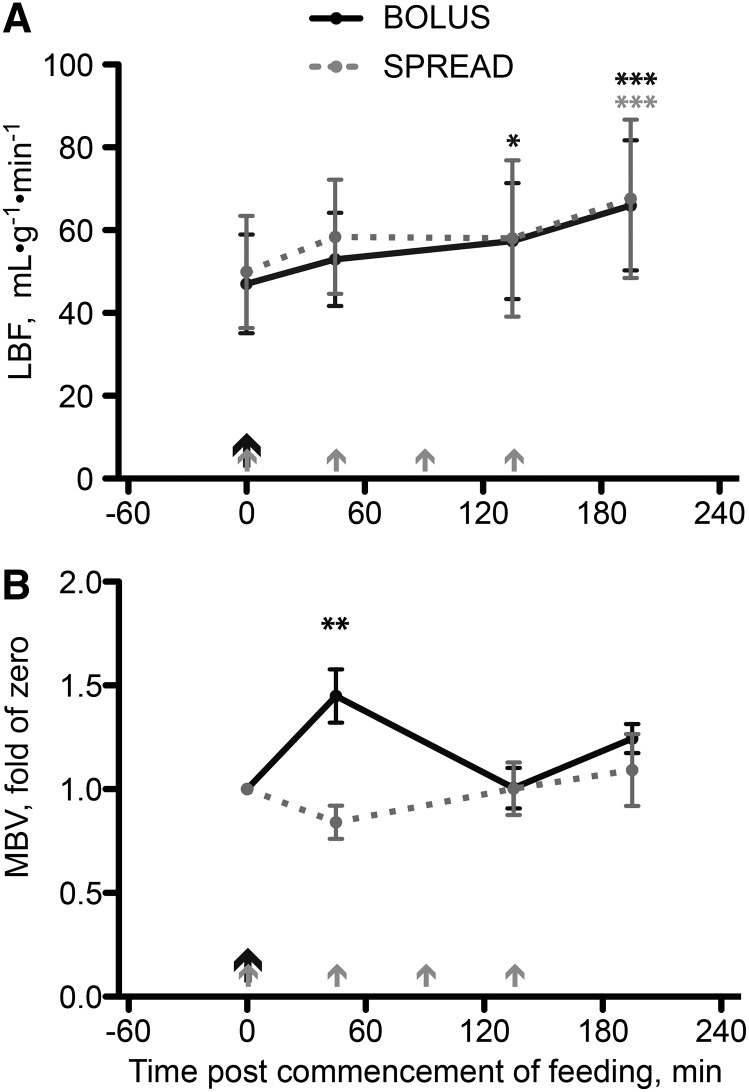
LBF (A) and MBV (B) in young men after consumption of 15 g of mixed-EAA doses by Bolus or Spread treatment. The black arrows represent ingestion of 15 g EAAs once, and the gray arrows represent ingestion of 3.75 g EAAs 4 times. Values are means ± SEMs, *n* = 8. *^,^**^,^***Different from fasted: *P < 0.05, **P < 0.01, ***P < 0.001. EAA, essential amino acid; LBF, leg blood flow; MBV, microvascular blood volume.

Fasting LBF was similar between groups (*P* = 0.7) and increased during postprandial periods (approximately +40% at 195 min; *P* < 0.001 vs. fasting for both) irrespective of feeding strategies (**Figure 4**A). Fasting muscle MBV was also similar between groups (Bolus: 0.90 ± 0.16 units · mm^−3^; Spread: 0.93 ± 0.12 units · mm^−3^; *P* = 0.85). Subsequent calculations were based on each individual standardized to his own fasting MBV. Upon Bolus feeding, MBV increased (+45% at 45 min; *P* = 0.0067 vs. fasting) and returned to fasted levels by 135 min postfeeding, demonstrating transient capillary recruitment with feeding. No change in MBV was seen after Spread feeding (Figure 4B). At 45 min postcommencement of feeding, the difference in MBV between strategies was also significant (*P* < 0.001).

#### Oral EAAs induced changes in established signaling pathways including mTORC1 substrates.

**FIGURE 5:**
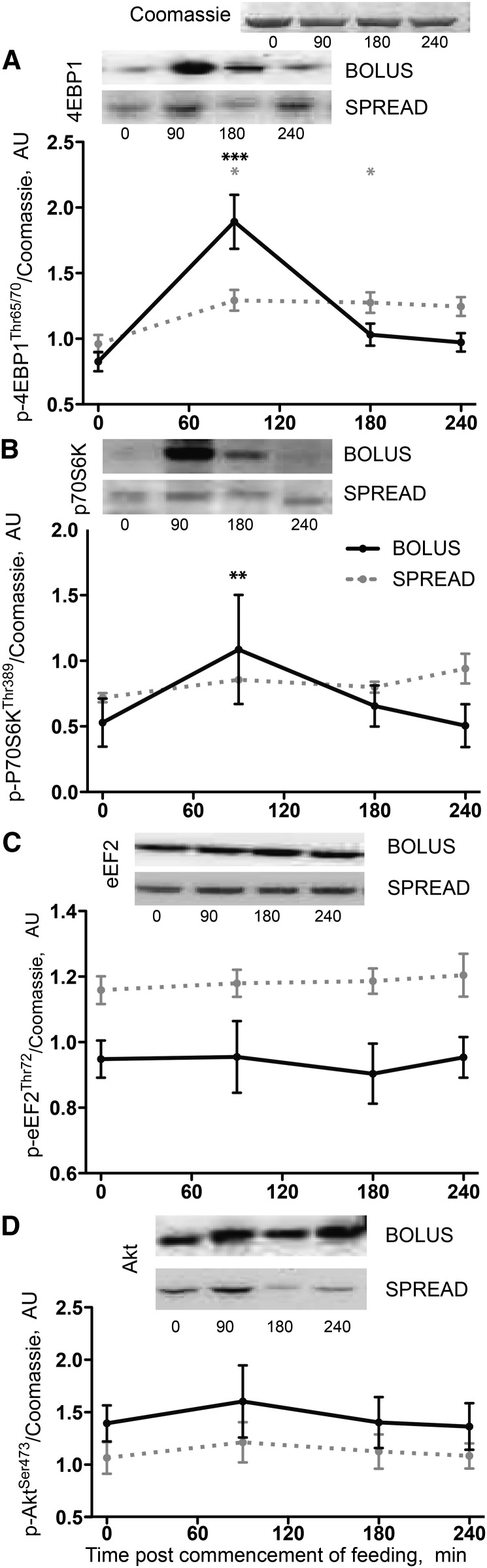
Phosphorylation of 4EBP1^Thr65/70^ (A), P70S6K^Thr389^ (B), eEF2^Thr56^ (C), and Akt^Thr473^ (D) in young men after consumption of 15 g of mixed-EAA doses by Bolus or Spread treatment. Values are means ± SEMs, *n* = 8. *^,^**^,^***Different from fasted: *P < 0.05, **P < 0.01, ***P < 0.001. Akt, protein kinase B; AU, arbitrary units; eEF2, eukaryotic elongation factor 2; p-, phosphorylated; P70S6K1, ribosomal protein S6 kinase 1; 4EBP1, eukaryotic translation initiation factor 4E-binding protein 1.

The profile of postprandial mTORC1 substrate phosphorylation differed between feeding strategies. Phosphorylation at 4EBP1^Thr65/70^ was increased at 90 min after Bolus feeding (+130%; *P* < 0.001 vs. fasting) but had returned to basal levels by 180 min, whereas with Spread feeding, the increase was smaller (+34% at 90 min; *P* = 0.021) but sustained (+32% and +30% at 180 and 240 min, respectively; *P* = 0.026 and 0.043, respectively; **Figure 5**A). A similar pattern was apparent in phosphorylation at phosphorylated (p)-p70S6K^Thr389^ (Bolus: +105% at 90 min; *P* = 0.002 vs. fasting), although increased noise-to-signal prevented detection of significant changes with Spread feeding (Figure 5B). Significant changes from fasting were not detected in p-Akt^Thr473^ or p-eEF2^Thr72^ (Figure 5C, D).

#### Oral EAAs induced changes in MPS and relations with plasma insulin, EAAs, and anabolic signaling.

**FIGURE 6:**
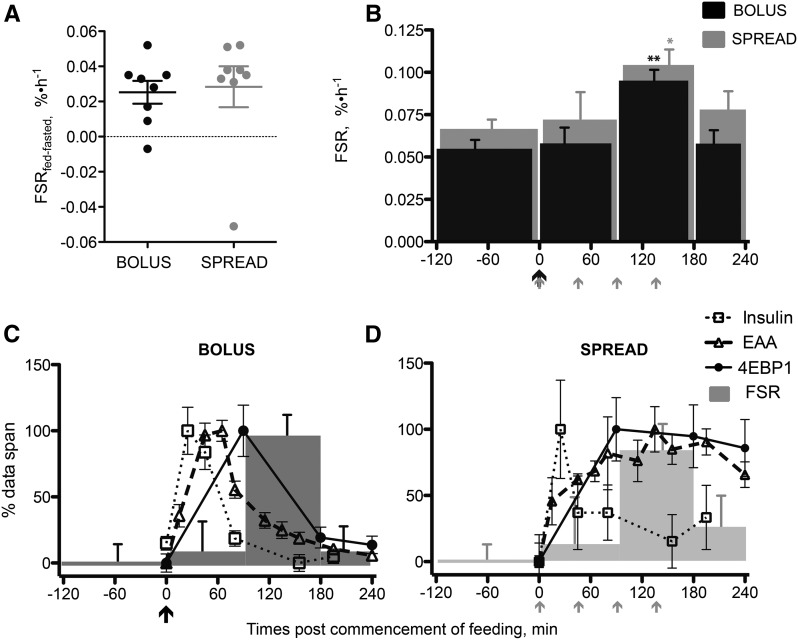
Absolute changes in FSR from fasted (−120 to 0 min) to fed (0 to 240 min) (A), actual FSRs (B) and plasma EAA and insulin concentrations, phospho-4EBP1^Thr65/70^ and muscle protein synthetic rates, normalized to their own data spans shown on the same axis (C and D) in young men after consumption of 15 g of mixed-EAA meals by Bolus or Spread treatment. The black arrows represent ingestion of 15 g EAAs once, and the gray arrows represent ingestion of 3.75 g EAAs 4 times. Values are means ± SEMs, *n* = 8. *^,^**Different from fasted: *P < 0.05, **P < 0.01. EAA, essential amino acid; FSR, fractional synthetic rate; 4EBP1, eukaryotic translation initiation factor 4E-binding protein 1.

The fasting FSR, measured across 2 h, was similar between groups (Bolus: 0.054 ± 0.005% · h^−1^; Spread: 0.066 ± 0.005% · h^−1^; *P* = 0.34). A similar postprandial increment in FSR, measured across 4 h, was observed regardless of feeding strategy (Bolus: 0.025 ± 0.006% · h^−1^; Spread: 0.028 ± 0.012% · h^−1^;* P* = 0.81) (**Figure 6**A). Detailed temporal resolution of the postprandial period revealed no group differences; neither Bolus nor Spread feeding altered FSRs during the period 0–90 min postfeeding. During the period 90–180 min postfeeding, the FSR increased with Bolus feeding (to 0.095 ± 0.006% · h^−1^; *P* = 0.0088 vs. fasting) as did Spread feeding (to 0.104 ± 0.009% · h^−1^; *P* = 0.013). In the period 180–240 min postcommencement of feeding, FSRs in both Bolus and Spread groups had returned to fasted rates (Figure 6B). To view temporal relations, plasma concentrations of insulin and EAAs, phosphorylation of 4EBP1^Thr65/70^ and MPS, normalized to their own data-spans, were placed on the same axis (Figure 6C, D).

## Discussion

We present novel data quantifying the temporal muscle anabolic response to a physiologically relevant oral EAA dose provided as a Bolus or Spread feeding strategy and contextualized this in terms of aminoacidemia/insulinemia, microvascular flow, and intramuscular signaling response profiles. Despite distinct plasma and muscle profiles, Bolus feeding provided no anabolic advantage over Spread feeding (or vice versa); these findings are in keeping with our hypothesis of there being an intrinsic muscle-full state in young men at rest.

Bolus feeding led to rapid aminoacidemia with a brisk upstroke and high peak plasma EAA and leucine concentrations. Spread feeding, by comparison, resulted in lower, later peak concentrations. Despite this, identical MPS responses were observed, even with the same latency (of ∼90 min) and amplitude. Furthermore, with both feeding strategies, basal MPS was observed 180 min after consumption of either Bolus or the initial Spread doses. This preceded the peak Spread plasma EAAs, in keeping with the onset of a muscle-full state. On this basis, our data suggest that, in healthy young men, it is dose-dependent mechanisms that regulate the size of the anabolic response to feeding and that this response is not perturbed by later arriving, lower-amplitude aminoacidemia. Because of the stability of muscle mass from year to year in healthy younger populations (29), it would seem vital to have such mechanisms in place to ensure adequate nourishment, given the irregularity of meals and variability in content, including slowly digested proteins (e.g., micellar casein).

Although identical temporal MPS responses to such divergent EAA availability profiles may be deemed unexpected, this can perhaps be explained by considering the postprandial period in 3 distinct phases. After the onset of essential aminoacidemia, a latent period exists when a significant negative arteriovenous EAA balance is detectable (21) but incorporation of EAAs into newly synthesized myofibrillar proteins is not. The existence of a similar latent period in response to Bolus and Spread EAA ingestion suggests that providing time for adequate intracellular EAA accumulation, even with rapid aminoacidemia with Bolus, is crucial before MPS can be “switched on.”

After this latent period, a transient stimulation in MPS, lasting ∼90 min (7), occurs before the onset of the muscle-full state restores basal MPS despite sustained, near-peak postprandial EAA availability (7–9). Should dose-dependent mechanisms underlie the onset of the muscle-full state (i.e., sufficient MPS was achieved with both ingestion strategies by 180 min), this would constrain stimulated MPS to the period 90–180 min post commencement of EAA ingestion and result in identical temporal patterns of anabolism. Indeed, our observation that both groups enter the muscle-full state while achieving an identical amount of MPS, despite markedly different patterns of EAA exposure highlights that “dose-dependent” mechanisms underlie the muscle-full effect (i.e., MPS responses are dependent on the amount of newly translated myofibrillar proteins rather than time-dependent mechanisms, triggered by EAA availability). We can be confident in these conclusions because repeated biopsies taken across the postprandial period provided us temporal resolution whereby only subtle and physiologically inconsequential differences in the relative duration of each phase of MPS after Spread and Bolus could be missed. A dose-dependent muscle-full mechanism that limits postprandial MPS may support the distribution of dietary protein across meals as a beneficial strategy (30).

The provision of free EAAs did not achieve a more rapid plasma appearance of EAAs or leucine than that previously observed with whey ingestion (9). Indeed, although one may intuitively expect free amino acids to be more rapidly absorbed, the rapidity of enzymatic peptide hydrolysis and H^+^-dependent di- and tripeptide transporters in the duodenum and proximal jejunum allow polypeptides such as leucine to acheive plasma aminoacidaemia after the same latency as monomeric amino acid ingestion. The period between plasma EAA appearance and MPS stimulation also varies with mode of provision; with primed-constant intravenous EAAs this was at least 30–45 min (7), whereas this period was 45–60 min after whey protein ingestion (9). Thus, stimulated MPS is observed 60–90 min postfeeding with an EAA bolus and 45–60 min with whey. Given the similarity in plasma EAA and leucine profiles between these different treatments, it would seem that an explanation for this difference may reside with pathways initiated in the gut rather than just at the myocyte and may be because of factors other than amino acid composition differences, which cannot be accounted for without identically matching and comparing the anabolic effects of whey with equivalent free amino acids. In addition, in studies so far, a lack in frequency of muscle sampling and/or a matching in timing of sampling has hampered identification of the specific reasons behind latency duration (4, 6, 12, 16, 31).

We observed a subtle increase in plasma insulin with Bolus feeding, reflecting the moderate insulin secretagogue properties of EAAs (32), an effect attenuated with Spread feeding. Identical MPS between groups further supports the hypothesis that postabsorptive insulin concentrations are compatible with maximal MPS (i.e., there is no additive effect of insulin over and above aminoacidemia alone) (26, 33–35). In terms of muscle microvascular blood flow, early (45 min after feeding) postprandial capillary recruitment (increases in MBV) was present only with Bolus feeding, in keeping with this being insulin mediated (19, 36). This phenomenon is thought to facilitate nutrient delivery by increasing the microvascular exchange surface area and decreasing the distancing from capillary to myocyte (21, 37). Thus, the difference between Bolus and Spread feeding in actual availability of EAAs to myocytes is underestimated by simple consideration of differences in plasma concentration. This points toward, at least in healthy young individuals, a redundancy in delivery of EAAs whereby postprandial muscle microvascular responses are not vital in achieving optimal MPS (38). In contrast to MBV, LBF increased similarly in both Spread and Bolus groups, suggesting that insulin-independent, EAA-mediated mechanisms affect bulk flow through the limb. This is supported by our previous observation of similar changes in LBF in response to EAA infusion across a wide range of clamped insulin concentrations (26). Divergent profiles of MBV and LBF highlight the inadequacy of measures of large vessel or whole-limb flow independently in assessing muscle microvascular changes (21).

Greater phosphorylation of mTOR substrates 4EPB1 and p70S6K in Bolus vs. Spread feeding confirms that rapid aminoacidemia enhances intramuscular anabolic signaling (12, 39), although this does not necessitate enhancement of MPS as there is an established dissociation between anabolic signaling and MPS (26). Although the stimulation of MPS by EAA feeding is blocked by rapamycin, showing that the effect requires mTORC1 activation (40), our data argue against phospho-activity of these pathways being proportional to increases in MPS. Interestingly, phosphorylation of 4EBP1^Thr65/70^ reflected substrate availability (Figure 6C, D).

In terms of possible limitations to this study, our conclusions may apply selectively to a young healthy population at rest and who receive an adequate, or maximal, high-quality diet. The delivery profile may affect the ability of a smaller feed, or one less abundant in leucine, to stimulate MPS. Divergence between young and old may depend on the ingested dose of EAAs (4, 35, 41, 42), highlighting the importance of separate consideration of feeding profile in elderly or sarcopenic individuals. The invasive nature of this study, with multiple biopsies, precluded a “crossover” design and necessitated termination before Spread plasma EAA and leucine returned to basal concentrations, contributing to apparently reduced incremental AUCs in that group. Historic studies that pointed toward rate of EAA appearance affecting anabolism were confounded by differing compositions, e.g., whey vs. casein or soy (11, 13, 14, 43). Perhaps significantly, recent studies supporting the notion that delivery profile affects MPS measured anabolism of nutrition in combination with resistance exercise (12, 16) or rapid growth (44). As such, different regulators of the size of the protein synthetic response to feeding are brought to bear in such periods of net muscle mass accretion.

To conclude, our data do not support the notion that rates of plasma aminoacidemia are a key driver of anabolism when young individuals (at rest) consume the same quantity of EAAs. Instead, the onset of the muscle-full state acts to limit MPS despite ongoing nutrient availability, further positioning this phenomenon as a key regulator of muscle protein homeostasis.

## Footnotes

6 Abbreviations used: AKT, protein kinase B; CEUS, contrast-enhanced ultrasound; EAA, essential amino acids; eEF2, eukaryotic elongation factor 2; FSR, fractional synthetic rate; LBF, leg blood flow; MBV, microvascular blood volume; MPS, muscle protein synthesis; mTORC1, mammalian target of rapamycin complex 1; p, phosphorylated; p70S6K1, ribosomal protein S6 kinase 1; 4EBP1, eukaryotic translation initiation factor 4E-binding protein 1.
